# Study of the Effect of Different Hepatitis C Virus Genotypes on Splenomegaly

**DOI:** 10.7759/cureus.10164

**Published:** 2020-08-31

**Authors:** Muhammad Junaid Azhar, Noor Khalid, Shanza Azhar, Umer Irshad, Hassaan Ahmed, Tayyab Mumtaz Khan, Samat Habib, Zuha Ali, Yumnah Anwar, Muhammad Bilal

**Affiliations:** 1 Internal Medicine, Rawalpindi Medical University, Rawalpindi, PAK; 2 Internal Medicine, Foundation University Medical College, Islamabad, PAK

**Keywords:** hcv, genotypes, splenomegaly

## Abstract

Background

Several recent studies have shown that the hepatitis C virus (HCV) and its different genotypes are a predominant and leading cause of cirrhosis and splenomegaly in different regions of the world. Advanced stage of cirrhosis leads to portal hypertension that causes splenomegaly. This complication may have many other manifestations such as anemia, infections, and bleeding disorders in severe stages. This study aimed to determine the effect of different HCV genotypes on the development of splenomegaly and to assess which HCV genotypes are more associated with it.

Materials and methods

A total of 483 conveniently sampled HCV patients were included in this descriptive cross-sectional study. Six genotypes (1, 2a, 2b, 3a, 3b, and mixed) were studied, and 80 patients for each of these genotypes were included. Data were collected from patient medical records regarding patient demographic details, HCV serology and genotyping, and sonographic size of the spleen.

Results

In total, splenomegaly was present in 14.1% (n=68) patients. The development of splenomegaly was significantly associated with old age, as 25.2% (n=26) of patients above 60 years of age (n=103) developed splenomegaly (P=0.005). Our study determined that splenomegaly was significantly related to HCV genotypes 3a, 3b, and 1 (P<0.001, P=0.017, and P=0.019, respectively). By taking mixed genotype as a reference, the odds of developing splenomegaly with genotype 3a were the highest (OR = 9.481; CI=95%).

Conclusions

Our study concludes that HCV genotype 3a, 3b, and 1, and age above 60 years have a significant association with splenomegaly. Genotype 3a has the highest risk of developing splenomegaly. Therefore, our study demands screening, early diagnosis, and prompt treatment of these particular HCV genotypes to prevent complications and risk of mortality.

## Introduction

Hepatitis C virus (HCV), a member of the *Flavivirus* group, is a significant cause of chronic liver disease and cirrhosis, particularly in developing countries like Pakistan. The cirrhotic liver causes a high portal venous pressure (PVP) and splenomegaly. An enlarged spleen can then lead to further complications such as hypersplenism, increased spleen thickness, infarction, and splenic rupture [1]. It is estimated that 177.5 million people worldwide (2.5% of the world population) are infected with hepatitis C [2]. In the Asia-Pacific region, the prevalence of HCV ranges from 0.1% to 4.7% [3]. Hepatitis C infection is particularly prevalent in Pakistan, with prevalence rates of approximately 6-7% nationwide and up to 15% in its most populous province Punjab [4,5].

Clinically, HCV is classified into seven major genotypes (1, 2, 3, 5, 6, 7, and mixed) and multiple distinct subtypes (a, b, c, etc.) [2,6]. Genotypes (GT) 1, 2, and 3 are responsible for the most cases of HCV worldwide. HCV GT 3 remains the predominant subtype in South Asia. The most prevalent HCV genotype in Pakistan is GT 3, especially its subtype 3a, which makes up 61.3% of all HCV patients in Pakistan [4]. GT 3 is also the most virulent subtype, with an accelerated disease progression to cirrhosis and a worse response to antiviral drugs [7].

Splenomegaly has a variable incidence in cirrhosis and occurs in its severe stages. The most widely accepted explanation for this phenomenon demonstrates that resistance to splenic vein outflow causes an increase in PVP, which leads to back-pooling of blood within the spleen and, consequently, its enlargement. Splenomegaly is associated with an overall poor prognosis in HCV patients [8].

It remains a well-known fact that the specific HCV genotype influences the clinical course and severity of the disease, as well as the response to antiviral therapy [9]. Hence, the HCV genotype and its virulence may have a direct implication on the development of splenomegaly later in the disease and may explain the overall variable rates of splenic enlargement. Studies have shown that GT 3 is associated with a faster progression to cirrhosis [7], and, therefore, we expect to have an increased incidence of splenomegaly in GT 3 patients. This would warrant increased surveillance and aggressive treatment of splenomegaly in such patients to avoid possibly life-threatening complications such as infections, anemia, bleeding, stiffness, and spleen rupture.

The relationship between HCV genotype and disease progression has been explored by several studies in the past [10]. However, studies examining the potential association of genotype with splenomegaly are extremely scarce. Our study aims to address this gap in the international scientific literature. We aim to evaluate the relationship between various HCV genotypes and sonographic spleen size. A significant association between these two parameters will have clinical and diagnostic applications and will help in the early diagnosis and management of HCV-related splenic complications.

## Materials and methods

Study population and design

This descriptive cross-sectional study was conducted from March 2018 to August 2018 at the Liver Centre of Holy Family Hospital, Rawalpindi, Pakistan. A sample of 483 participants (259 males and 224 females) was taken. Data of the 483 naïve HCV patients (diagnosed for the first time) were obtained from hospital records. These patients were sampled conveniently regardless of age and gender. Six HCV genotypes were studied, including GTs 1, 2a, 2b, 3a, 3b, and mixed genotype (two different genotypes present in the same patient). For each genotype, an approximately equal number of patients (n=80) were selected. Patients with other co-morbid conditions that can cause splenomegaly (e.g., malaria, hematological malignancies such as leukemia and lymphoma, and hemolytic anemias) were excluded. Categorical variables studied retrospectively were age, gender, HCV genotype, and splenomegaly.

Assessment tools

We obtained data regarding demographic details and the results of laboratory tests performed for the detection of HCV from patient medical records. According to hospital records, qualitative and quantitative tests for hepatitis C had been performed for all patients at the time of admission. HCV seropositivity had been confirmed through qualitative anti-HCV IgG (immunoglobulin G) assay (ELISA testing) using the HCV EIA 2.0 kit (Abbott Laboratories, Des Plaines, IL, USA) [11]. All patients had undergone quantitative PCR analysis for HCV RNA using the Abbott™ RealTime HCV assay (Abbott Laboratories) [11]. HCV genotyping had been carried out via the Versant™ HCV Genotype Assay LiPA (version I, Siemens Medical Solutions, Diagnostics Division, Fernwald, Germany), which detects HCV genotypes based on the 5’UTR (5′ untranslated region) sequence [12]. The results of these tests were noted for every patient included in our study through their records. A routine abdominal ultrasound was performed for all patients during their hospital stay, and we used the reports to assess the size of the spleen. For all patients, we defined splenomegaly as a craniocaudal length of greater than 13 cm, in accordance with other studies [13].

Statistical analysis

All statistical analyses were carried out using the statistical software SPSS Version 22.0 for Windows (IBM Corp., Armonk, NY, USA). Using descriptive statistics, we determined the frequencies and percentages of the distribution of categorical variables in the study population. The association of HCV genotypes with splenomegaly was determined using binary logistic regression test, and the risk of developing splenomegaly in a specific genotype was determined by calculating odds ratios (ORs) with a 95% confidence interval (CI). A P-value of less than 0.05 was considered significant. We then performed cross-tabulation between genotypes and splenomegaly to determine which HCV genotype has a higher association with splenic enlargement.

## Results

The demographic characteristics of the patients are shown in Table 1. Most of the patients (n=238, 49.3%) were in the age group of 41-60 years, whereas 29.4% of patients (n=142) belonged to the age group of 20-40 years. There were 21.3% (n=103) patients in the age group above 60 years of age. The majority of the patients were males (53.6% [n=259]). Females made up 46.4% (n=224) of the study population.

**Table 1:** Age and gender distribution of the study population.

Category	Frequency	Percentage
Age		
20-40 years	142	29.4
41-60 years	238	49.3
>60 years	103	21.3
Gender		
Male	259	53.6
Female	224	46.4

Splenomegaly was present in 14.1% (n=68) of the patients; 85.9% (n=415) patients had no splenomegaly at the time of the study (Table 2).

**Table 2:** Presence of splenomegaly in the study population.

	Frequency	Percentage
Splenomegaly present	68	14.1
Not present	415	85.9
Total	483	100.0

Table 3 shows the adjusted ORs for GT 1 (OR=4.278; 95% CI: 1.273-14.383), GT 3a (OR=9.481; 95% CI: 3.082-29.1667), and GT 3b (OR=4.290; 95% CI: 1.297-14.187). These three genotypes (GTs 1, 3a, and 3b) were significantly associated with splenomegaly when compared to mixed genotype, which was taken as reference (P=0.019, P<0.001, and P=0.017, respectively). Compared with the age group of 20-40 years, which was taken as reference, the age group above 60 years of age was significantly associated with a higher frequency of splenomegaly (OR=3.194; 95% CI: 1.417-7.20; P=0.005). The effect of gender on splenomegaly was not significant.

**Table 3:** Binary logistic regression analysis. Variables entered are genotype, age, and gender of the patient.

Variables	Splenomegaly present		Splenomegaly absent		Sig.	Adjusted odds ratio	95% confidence interval	
	n	%	n	%			Lower	Upper
Genotype 1	12	15%	68	85%	0.019	4.278	1.273	14.383
Genotype 2a	7	8.6%	74	91.4%	0.137	2.704	0.729	10.036
Genotype 2b	4	4.9%	78	95.1%	0.987	1.012	0.240	4.271
Genotype 3a	28	35%	52	65%	<0.001	9.481	3.082	29.166
Genotype 3b	13	16.2%	67	83.8%	0.017	4.290	1.297	14.187
Genotype mixed	4	5%	76	95%	Reference			
Age 41-60 years	30	12.6%	208	87.4%	0.478	1.308	0.622	2.749
Age >60 years	26	25.2%	77	74.8%	0.005	3.194	1.417	7.200
Age 20-40 years	12	8.5%	130	91.5%	Reference			
Male	29	11.2%	230	88.8%	0.175	0.663	0.366	1.200
Female	39	17.4%	185	82.6%	Reference			

We used expected count and standardized residual to determine which HCV genotypes were significantly associated with a higher prevalence of splenomegaly, and found that GT 3a had a higher association with splenomegaly. Out of a total of 68 (14.1%) patients who developed splenomegaly, 28 (35%) patients belonged to GT 3a. The next most common genotypes were GT 3b (n=13 [16.2%]) and GT 1 (n=12 [15%]). Mixed genotype showed the least association with splenomegaly (n=4 [5%]). These results are represented in Table 4.

**Table 4:** Genotypes and Splenomegaly Cross-Tabulation.

Genotypes			Splenomegaly		Total
			Yes	No	
	Genotype 1	Count	12	68	80
		Expected count	11.3	68.7	80.0
		% within genotype	15.0%	85.0%	100.0%
		% within splenomegaly	17.6%	16.4%	16.6%
		% of total	2.5%	14.1%	16.6%
		Standardized residual	0.2	-0.1	
	Genotype 2a	Count	7	74	81
		Expected count	11.4	69.6	81.0
		% within genotype	8.6%	91.4%	100.0%
		% within splenomegaly	10.3%	17.8%	16.8%
		% of total	1.4%	15.3%	16.8%
		Standardized residual	-1.3	0.5	
	Genotype 2b	Count	4	78	82
		Expected count	11.5	70.5	82.0
		% within genotype	4.9%	95.1%	100.0%
		% within splenomegaly	5.9%	18.8%	17.0%
		% of total	0.8%	16.1%	17.0%
		Standardized residual	-2.2	0.9	
	Genotype 3a	Count	28	52	80
		Expected count	11.3	68.7	80.0
		% within genotype	35.0%	65.0%	100.0%
		% within splenomegaly	41.2%	12.5%	16.6%
		% of total	5.8%	10.8%	16.6%
		Standardized residual	5.0	-2.0	
	Genotype 3b	Count	13	67	80
		Expected count	11.3	68.7	80.0
		% within genotype	16.2%	83.8%	100.0%
		% within splenomegaly	19.1%	16.1%	16.6%
		% of total	2.7%	13.9%	16.6%
		Standardized residual	0.5	-0.2	
	Mixed genotype	Count	4	76	80
		Expected count	11.3	68.7	80.0
		% within genotype	5.0%	95.0%	100.0%
		% within splenomegaly	5.9%	18.3%	16.6%
		% of total	0.8%	15.7%	16.6%
		Standardized residual	-2.2	0.9	
Total		Count	68	415	483
		Expected count	68.0	415.0	483.0
		% within genotype	14.1%	85.9%	100.0%
		% within splenomegaly	100.0%	100.0%	100.0%
		% of total	14.1%	85.9%	100.0%

## Discussion

HCV is one of the leading causes of morbidity and mortality in the world today. It causes progressive chronic inflammation and tissue damage in the liver. The process of hepatic fibrosis ensues in response to this tissue injury and ultimately results in end-stage cirrhosis [14]. The incidence of the development of cirrhosis in HCV patients is around 20% after 20 years of infection [15]. This high incidence of cirrhosis is especially concerning for developing countries like Pakistan, where the rates of hepatitis C infection are much higher than in the West [16].

The cirrhotic liver causes various complications including hepatic decompensation and development of hepatocellular carcinoma. These complications are often fatal, and hence it is essential to diagnose and treat HCV early [9]. Hepatitis C also has numerous extra-hepatic manifestations. Portal hypertension, which arises as a result of the liver cirrhosis, manifests itself as esophageal varices, ascites, and splenomegaly at different stages of the disease. This study is an effort to establish a relation between various HCV genotypes and spleen enlargement and also defines the impact of age on the development of splenomegaly.

Splenomegaly is a common finding in end-stage cirrhotic patients. In our study, descriptive statistics showed that around 14.1% (n=68) HCV patients developed splenic enlargement as a result of their cirrhosis. Our findings are similar to a recent study published in 2017, which reports that the incidence of hypersplenism and splenomegaly in cirrhotic patients ranges from 11% to 55% [17].

Splenomegaly commonly occurs in the late and severe stages of cirrhosis and is easily detectable on abdominal examination and ultrasound. Hence, it can serve as a non-invasive assessment tool for the detection of the severity of the disease process. The presence of an enlarged spleen in a hepatitis C patient would suggest that the disease has now progressed to liver cirrhosis, and it is a poor prognostic marker. In our study, we used splenomegaly as a non-invasive marker to detect the effect of various factors such as age, gender, and the genotype of the virus on disease progression.

In cross-tabulation between HCV genotypes and splenomegaly, out of all 68 patients (100%) who developed splenomegaly, GT 3a was the most common (41% [n=28]). The incidence of splenomegaly was 35% in GT 3a, 16.2% in GT 3b, and 15% in GT 1. These findings are in accordance with a study conducted in Brazil that showed the incidence of splenomegaly in GT 3 to be much higher than its incidence in GT 1. In that study, Castro et al. found the incidence of splenomegaly in GT 3 (both subtypes included) to be 30%, which is close to our findings [18].

Gupta et al. showed that GT 3 was associated with an increased rate of progression to cirrhosis due to enhanced interferon gene stimulation in non-parenchymal cells [8]. Another study conducted across different provinces of China showed that GT 3b had a more rapid disease progression in Chinese patients [11]. In contrast, our study showed that GT 3a had a greater incidence of cirrhotic liver disease and splenomegaly.

In binary logistic regression analysis, our study showed that splenomegaly was significantly related to HCV GTs 3a, 3b, and 1 (P<0.001, P=0.017, and P=0.019, respectively). Out of all patients who developed splenomegaly, the least number of patients belonged to mixed genotype. By taking mixed genotype as a reference, the odds of developing splenomegaly with GT 3a were the highest (OR = 9.481; CI=95%). The second and third in line were GT 3b (OR=4.290; CI=95%) and GT 1 (OR=4.278; CI=95%), respectively.

Our findings report that GT 3, along with its subtypes GT 3a and GT 3b, shows the highest correlation with splenomegaly among all genotypes. This is also an indirect measure of virulence: a stronger association with splenomegaly (a complication of cirrhosis) suggests a more progressive course of the disease. GT 3 is also the most common in Pakistan [4].

Lu et al. showed that GT 3 was associated with 42.2% of cirrhotic patients compared to 27.0% non-cirrhotic patients. The stage of fibrosis, as measured by APRI (aspartate aminotransferase-to-platelet ratio index), was higher in younger patients with GT 3 (below 50 years old). This study explained disease progression by estimating that the disease duration for developing cirrhosis was 16.6 years for GT 3 and 21.0 years for non-GT 3 (P=0.04) [7]. The earlier the cirrhosis develops, the more likely splenomegaly will develop in the course of the HCV genotype. Our study results also prove that GT 3 is more associated with spleen enlargement.

We also evaluated the variation of prevalence of splenomegaly with the age of study participants. By dividing all patients into three age groups, 20-40, 41-60, and above 60 years, patients with age above 60 years showed a significant prevalence of splenomegaly (P=0.005). In this age group, 26 (25.2%) out of 103 (74.8%) patients developed splenomegaly. Odds of having splenomegaly above age 60 were 3.194 (95% CI: 1.417-7.20; P=0.005) times higher as compared to the odds of developing splenomegaly in the 20-40 age group, which was taken as a reference because the least number of patients in this group showed splenomegaly. Our findings explain that late presentation and delayed diagnosis of hepatitis C in older patients is associated with the development of more complications such as splenomegaly. Elderly patients also show a poor response to interferon treatment and are more resistant to achieve a high rate of sustained virologic response [19]. The effect of gender was not significant. Male and female populations showed approximately equal chances of having splenomegaly.

Literature review did not reveal studies that explore the basic idea behind this research. To the best of our knowledge, there is no study in the local or international scientific literature that exactly corresponds with our study. Based on our findings, we can draw two conclusions. Firstly, GTs 3b, 1, and especially 3a, are more likely to develop splenomegaly and cirrhosis. The most probable explanation for this fact is the late presentation and diagnosis in such patients. Secondly, GT 3a may be more resistant to treatment by DAAs (direct-acting antivirals) and hence it may have more chances of disease progression and splenomegaly. GT 3 viruses also have a low sensitivity against DAAs and show more association with steatosis [7]. In accordance with our statistics, our study calls for earlier screening and diagnosis of hepatitis C infection and the detection of its genotypes. Early treatment of GTs 3a, 3b, and 1 can prevent hepatic complications and reduce the mortality risk.

The main limitation of our study is that we did not include the patients belonging to HCV GTs 4, 5, 6, and 7. These genotypes were rare at the time of our study, and we did not find an ample number of patients for these genotypes.

## Conclusions

Splenomegaly was present in 14.1% of all hepatitis C patients, and the highest prevalence was observed in GT 3a. Other genotypes significantly associated with splenomegaly were GTs 3b and 1. Patients diagnosed in old age, especially above age 60, seem to be the most susceptible to develop splenic enlargement. Hence, we recommend that efforts should be directed toward early screening and prompt diagnosis of these HCV genotypes. Treatment should be commenced earlier in these patients to avoid the development of complications such as splenomegaly, which serve as a poor prognostic marker in the course of the disease.
